# Relationships Among Mobile Internet Use, Social Support, and Depressive Symptoms: Prospective Cohort Study Among Community Residents

**DOI:** 10.2196/76567

**Published:** 2025-11-14

**Authors:** Yingyue Xu, Meiqi Wang, Qixiu Li, Xiaoying Su, Long Sun

**Affiliations:** 1 Department of Social Medicine and Health Management, School of Public Health, Cheeloo College of Medicine, Shandong University, Jinan, China; 2 National Health Commission of China (NHC) Key Laboratory of Health Economics and Policy Research, Shandong University, Jinan, China; 3 Centre for Health Management and Policy Research, Shandong University (Shandong Provincial Key New Think Tank), 44 Wenhuaxi Road, Jinan, 250012, China, 86 13256660556; 4 School of Psychiatry and Psychology, Cheeloo College of Medicine, Shandong University, Jinan, China

**Keywords:** mobile internet use, social support, depressive symptoms, LASSO regression, cross-lagged model, least absolute shrinkage and selection operator

## Abstract

**Background:**

In the digital era, mobile internet integration into daily routines presents a paradoxical relationship with mental health outcomes. While previous cross-sectional studies report inconsistent associations between mobile internet use (MIU) and depressive symptoms, the longitudinal mechanisms involving social support remain underexplored.

**Objective:**

This 2-wave longitudinal study aimed to examine the temporal relationships between MIU, social support, and depressive symptoms among rural Chinese residents. Specifically, we hypothesize that (1) increased MIU will predict improved perceived social support over time, and (2) enhanced social support will mediate the relationship between MIU and reduced depressive symptoms. The findings are intended to inform digital health strategies that leverage internet-based interactions to improve mental well-being in underserved communities.

**Methods:**

A 2-wave longitudinal cohort study (4 y interval) was conducted among rural residents in Taierzhuang District, China (n=489 retained, mean age 63.30, SD 13.35 y; 310/489, 63.39% female). Multidimensional assessments included (1) demographic characteristics and MIU patterns via a customized survey, (2) social support was assessed using the Perceived Social Support Scale, and (3) depressive symptom severity via Center for Epidemiological Studies-Depression Scale. Advanced analytical strategies were implemented: least absolute shrinkage and selection operator regression for high-dimensional variable selection, complemented by cross-lagged panel modeling to disentangle temporal dependencies.

**Results:**

Our analysis found that the increase in MIU at baseline indicated the improvement of social support at follow-up (ρ=0.097, 95% CI 0.001-0.193; *P*=.049). There is a bidirectional cross-lagged relationship between social support and depressive symptoms. An increase in baseline social support indicates a reduction in later depressive symptoms (ρ=−0.096, 95% CI −0.183 to −0.01; *P*=.03). An increase in baseline depressive symptoms indicates a decrease in later social support (ρ=−0.109, 95% CI −0.213 to −0.004; *P*=.04). Baseline social support has a significant impact on later depressive symptoms. Further analysis revealed that social support fully mediated the relationship between MIU and depressive symptoms. There was no direct effect between the 2 stages of MIU and depressive symptoms.

**Conclusions:**

This study used longitudinal data and developed cross-lagged models to improve the reliability of findings and applied least absolute shrinkage and selection operator regression to improve the explanatory power and predictive accuracy of the models. The findings offer practical insights for designing digital mental health interventions, particularly in underserved rural areas. Specifically, our results support the development of mobile-based platforms that facilitate meaningful internet-based social interactions to bolster perceived social support and thereby reduce depressive symptoms. We recommend that public health initiatives incorporate digital literacy training and promote internet-based behaviors that strengthen real-life social connections. Furthermore, mental health professionals should consider individuals’ internet use patterns when designing personalized intervention strategies.

## Introduction

### Background

The current society has also entered a new era of digitalization, and the internet penetration rate is rising every year [1]. According to the Statistical Report on Internet Development in China, the number of netizens has grown from 620,000 in 1997 to 1.108 billion in 2024, and the internet penetration rate has risen to 78.6% [2]. The internet has gradually penetrated all aspects of personal life, and because mobile phones are powerful and widely used, they have had an important impact on people’s lives. Some studies have found that it has changed the way people consume information, reshaped people’s communication mode [3], facilitated people’s daily life, exacerbated the digital divide [4], and even had an impact on people’s mental health [5-10].

Related studies have found a significant association between mobile internet use (MIU) and depressive symptoms [11-14]. Notably, some studies have suggested that internet use, even for health purposes, may be associated with increased rates of depressive symptoms [15]. In addition, internet addiction and unhealthy internet-based behavior patterns (eg, excessive passive scrolling, exposure to cyberbullying, or engagement in upward social comparison) have been shown to exacerbate depressive symptoms in adolescent populations [16]. However, in a broader social context, for adults in general, the judicious use of the mobile internet—characterized by purposeful activities such as maintaining meaningful social connections, accessing evidence-based health information, and participating in supportive internet-based communities—appears to provide relief from depressive symptoms [11-14]. For older people in particular, the available evidence suggests that the internet may have a more positive impact. This may be due to the enhancement of their social connections and support networks through internet-based communication, for example, which indirectly reduces depressive symptom levels [12,17,18]. Although cross-sectional studies have repeatedly validated these ideas, there remains a relative paucity of research on the long-term relationship between the two [19]. Therefore, more longitudinal studies are needed to further explore how the general population can prevent or alleviate depressive symptoms through appropriate MIU.

Social support plays an important explanatory role in the negative correlation between mobile internet access and depressive symptoms. First, there is a strong link between MIU and social support [20-25]. For example, a Finnish study noted that cell phones can provide users with information and emotional support [22]. Another survey covering 7 European countries also showed that the use of the internet promotes the establishment of social support networks and improves the subjective health of individuals compared with other devices. In addition, several studies have found that the internet has become an important tool for maintaining and enhancing interpersonal relationships, and that people gain the necessary support and satisfaction from internet-based social interactions. On the other hand, social support is widely recognized as one of the key factors in maintaining an individual’s mental health [1]. A large body of literature shows that a good social support system is associated with lower rates of depressive symptoms [26-28]. Conversely, when a person lacks strong social support, they experience fewer positive emotions and their overall mental health declines, which increases the risk of depressive symptoms. These findings emphasize the importance of building a healthy social support environment for the prevention and treatment of depressive symptoms.

Many studies examining the association between MIU and depressive symptoms are limited by their design. Most rely on cross-sectional data [29], which cannot establish longitudinal relationship or determine whether frequent MIU leads to depression or vice versa. Furthermore, inconsistent definitions and measurements of social support across studies hinder the comparability and generalizability of findings [30,31]. Although recent longitudinal cohort studies have begun to clarify temporal relationships [19,32], many still lack comprehensive models and diverse populations.

### Objectives

**Figure 1.**
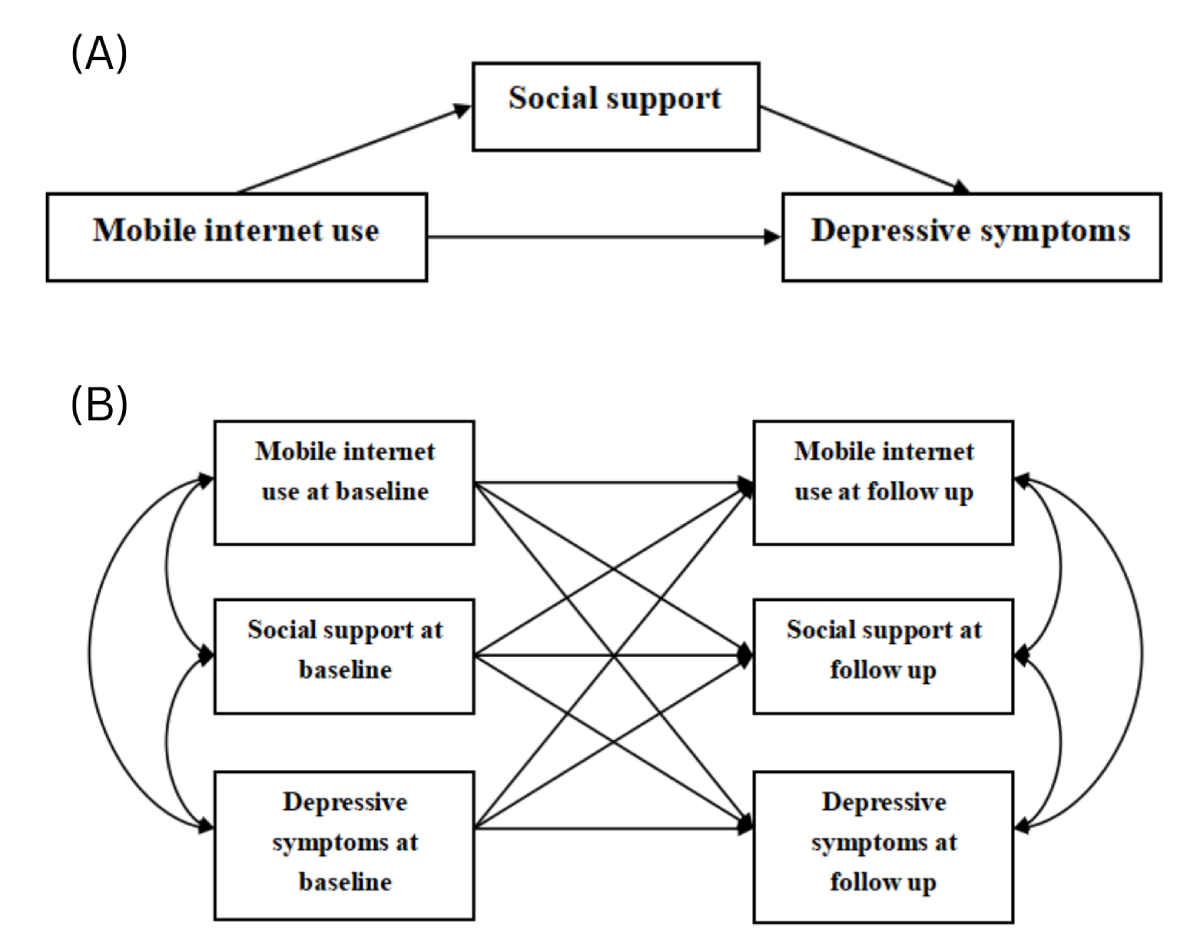
Analytical models of mobile internet use, social support, and depressive symptoms: (A) mediation model, and (B) cross-lagged panel model.

To address these gaps, our study uses a longitudinal design to examine the long-term effects of changes in MIU behavior on individuals’ social support and depressive symptoms. It uses analytical methods such as the least absolute shrinkage and selection operator (LASSO) regression and cross-lagged models to distinguish the mental health impacts of different usage patterns. Leveraging internationally recognized scales and multistage population surveys, the study systematically analyzes the longitudinal mechanisms and mediating pathways linking mobile internet usage, social support, and depressive symptoms to enhance the generalizability and reliability of findings. Ultimately, it aims to provide scientific evidence and intervention strategies for promoting healthy MIU, strengthening social support, and alleviating depressive symptoms. The conceptual framework and mediation model are presented in Figure 1.

## Methods

This prospective cohort study was reported in accordance with the Strengthening the Reporting of Observational Studies in Epidemiology (STROBE) guideline [33].

### Study Design

This longitudinal study used data collected across 2 waves (2019 and 2023) from rural residents in Taierzhuang District, Zaozhuang City, Shandong Province, China. Using a random cluster sampling approach, we selected 1 village from each of the 3 towns in Taierzhuang District, then recruited residents aged 18 years and older from these areas.

### Participants

The baseline survey (wave 1) was conducted in November 2019 and included 879 participants. The follow-up survey (wave 2) was administered in August 2023. Among the original participants, 51 had passed away, 145 had relocated away from the village, and 194 could not be contacted or declined to participate in the follow-up. Ultimately, 489 rural residents provided complete responses in both waves, representing the final sample for this 4-year longitudinal analysis.

### Data Collection

The implementation process of the 2023 Longitudinal Survey of Rural Residents is as follows. First, we communicated with and obtained support from doctors at local township health centers. We fully explained the survey content to residents beforehand to ensure voluntary participation and obtain signed informed consent forms (illiterate residents were assisted by their legal guardians). Second, trained graduate students conducted standardized one-on-one face-to-face surveys to ensure accurate questionnaire administration; during data cleaning, we identified minimal missing data, with only the follow-up social support scale having 6 missing values (1.23% of observations). Little’s Missing Completely at Random (MCAR) test was conducted (*χ*^2^_0_<0.001), which supports the conclusion that the data were missing completely at random. Given the low missing rate and MCAR pattern, we imputed these missing values using the mean of the available social support scores. All other variables had complete data. The entire process followed the principle of informed consent, and data quality was ensured through standardized procedures.

### Ethical Considerations

This research protocol received ethical approval from the Institutional Review Board at the Shandong University School of Public Health (Approval LL20190802). Written informed consent was obtained from all participants before enrollment. In cases involving illiterate or semiliterate individuals, consent was secured from their legal guardians. All data collected were anonymized through the use of unique identification codes. In compliance with institutional review board policies, participants did not receive financial compensation to prevent any potential coercion. The manuscript includes no personally identifiable information, and no images necessitating additional consent are present. The study was conducted in strict accordance with the ethical principles outlined in the Declaration of Helsinki and relevant Chinese regulations pertaining to research involving human participants.

### Measurement

#### Sociodemographic Variables

In this survey, the demographic variables of sex, age, ethnicity, marital status, education level, occupation, height, and weight were collected. Sex was measured as male (1) and female (0). According to the definition of the World Health Organization, age is divided into the non–older adult population group (≤60 y old) and the older adult population group (>60 y old). The ethnicity was measured by Han Chinese (1) and others (0), and the marriage was measured by married (1) and others (0). The education level is measured by illiteracy (1), primary school (2), and junior high school (3), and the occupation is measured by farmers (1) and others (0). BMI was calculated as weight in kilograms divided by height in meters squared (kg/m²).

#### MIU

MIU was measured using the following two questions, drawing upon previous literature [34]: “Do you go online?” The answers are divided into 3 categories: “independent internet access,” “internet access with the help of others,” and “no.” If someone answers “independent internet access” and “internet access with the help of others,” they need to answer the next question, “What are your internet tools?” The answer is set to multiple topics, including “desktop computer,” “laptop,” “tablet,” “mobile phone,” and “other devices.” If the answer option includes the “mobile phone” option, it is defined as “mobile internet user” (1); if not, it is defined as “other”(0).

#### Social Support

The Perceived Social Support Scale is used to assess the level of social support in this study [35]. This scale consists of 12 questions, each with 7 answers: “strongly disagree,” “disagree,” “slightly disagree,” “neutral,” “agree,” “slightly agree,” and “strongly agree.” The answer scores correspond to 1‐7 points. It includes 3 dimensions, namely “family support,” “friend support,” and “others support” [35]. Each dimension contains 4 questions, with a score of 4‐28 and a total score of 12‐84. The higher the score, the higher the social support. The Perceived Social Support Scale is currently a very popular and widely used scale internationally, with excellent reliability and validity. In previous studies, the Cronbach α coefficient for this scale was 0.96 [36]. In this study, the Cronbach α coefficients for the scale are 0.90 (wave 1) and 0.92 (wave 2), indicating excellent reliability.

#### Depression Symptoms

The Center for Epidemiological Studies-Depression Scale (CES-D) was used in this survey, specifically designed to evaluate the frequency of current depressive symptoms, with a focus on depressive emotions or mood [37]. It is important to note that the CES-D serves as a screening tool rather than a diagnostic instrument. This scale consists of 20 questions, reflecting 4 aspects of depressive symptoms: depressed affect, positive affect, somatic problems and retarded activity, and interpersonal relationship problems [37]. The answer includes: “occasionally or none (<1 d),” “sometimes (1‐2 d),” “frequent or half the time (3‐4 d),” and “most of the time or continuously (5‐7 d).” Respondents choose the appropriate answer based on their actual situation and frequency of feeling in the past week. Each question is assigned a score of 0‐3 points, with 4 items indicating nondepression and requiring reverse scoring. The total score range is 0‐60 points, and the higher the score, the higher the frequency of depression occurrence. For interpretive context, a score of 16 or higher is widely used in screening settings to indicate a possible depressive state. Referencing other studies on Chinese residents, the Cronbach α coefficient for this scale is 0.895 [38]. In this study, the Cronbach α coefficients for the scale are 0.91 (wave 1) and 0.90 (wave 2), indicating excellent reliability.

### Statistical Methods

#### Quality Control and Single Factor Analysis

After the survey was completed, EpiData software (version 3.1; EpiData Association) was used to input the paper data obtained from the survey into a computer, forming an electronic version of the data. Adopting a dual input format ensures the accuracy of data input, followed by data cleaning and analysis. Descriptive analysis and univariate analysis were conducted on demographic variables and core variables.

#### Multifactor Analysis Based on LASSO Regression

LASSO regression can solve the problem of multicollinearity, and its core idea is to reduce unimportant coefficients to 0 by introducing a penalty function. This algorithm not only achieves the visibility of model parameters, but also plays a role in feature selection [39].

LASSO regression analysis was conducted with R software (version 4.4.2; R Core Team) to control demographic variables and test the relationship between MIU, social support, and depressive symptoms. This version is widely used in medical data and statistics [40,41]. Model 1 examines the association between baseline MIU and follow-up depression, controlling for demographic variables. Model 2 adds baseline social support to test its potential mediating role between MIU and depression. Model 3 evaluates the relationship between baseline MIU and follow-up social support, controlling for covariates.

#### Robustness Analysis

To ensure the reliability of the LASSO regression results, we conducted comprehensive robustness analyses comprising bootstrap stability assessment with 1000 replications to evaluate variable selection consistency, complemented by sensitivity analysis examining variable selection patterns across different regularization parameters (including λ.min, λ.1se, and modified penalty strengths of 0.5×λ.min and 2×λ.min). Variables demonstrating selection frequencies exceeding 70% across these analyses were considered to exhibit high stability, providing robust evidence for the consistency of our identified predictors.

#### Analysis of Cross-Lagged Effect

We used Mplus (version 8.3; Muthen & Muthen) to conduct a cross-lagged panel model analysis, controlling for age, BMI, sex, ethnicity, marital status, occupation, and education. The model examined the reciprocal relationships between MIU, social support, and depressive symptoms across the 2 waves of measurement, and all path coefficients were analyzed. *P*<.05 indicates statistical significance. Mplus 8.3 offers advanced capabilities for handling complex models and missing data, providing greater flexibility in modeling scenarios where other software may not fully recognize the model. Using Mplus 8.3 ensures the reliability of results [42]. Finally, the cross-lagged model between MIU, social support, and depressive symptoms was established. The path coefficient ρ reflects the strength and direction of the longitudinal relationship between variables across time points. A significant cross-lagged path coefficient (eg, from baseline MIU to follow-up social support) suggests a longitudinal relationship in that direction. The relative magnitude and significance of the reciprocal paths (eg, social support to depression vs depression to social support) are compared to determine bidirectional or unidirectional relationships and determine whether variables have a 1-way or 2-way impact. If it is bidirectional, the degree of influence can be determined [43].

## Results

### Participants

**Figure 2.**
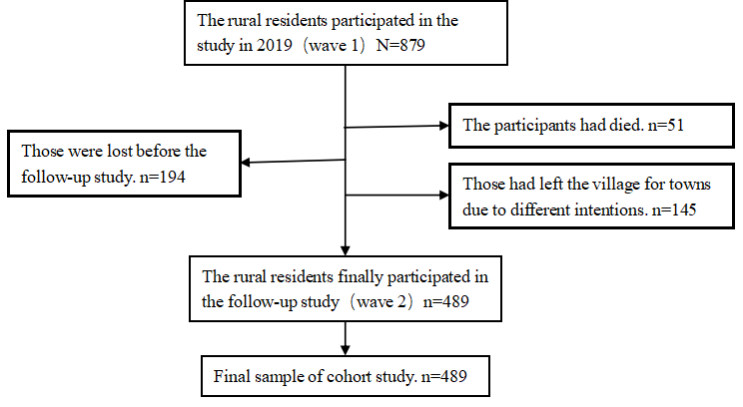
Inclusion and exclusion of rural resident samples standard.

In this longitudinal study, 489 rural residents successfully completed a questionnaire survey (mean age 63.30, SD 13.35 y; n=310, 63.39% female). Among them, the average age of the nonolder adult population group (age ≤60 y) was 50.00 (SD 8.82) years, while the average age of the older adult group (age >60 y) was 72.36 (SD 6.61) years. The participant flow is summarized in Figure 2. Table 1 describes the sample distribution of social demographics, MIU, social support, and depressive symptoms. The number of mobile internet users at baseline was 110, accounting for 22.49%, while the number of people who did not know how to use the internet was 379, accounting for 77.51%. The average scores of the social support scale at baseline and follow-up were 59.15 (SD 14.39) and 59.94 (SD 14.97), respectively. The average scores of the depression scale at baseline and follow-up were 10.39 (SD 10.59) and 10.74 (SD 11.19), respectively.

**Table 1.** Sample description and univariate analysis about the factors associated with depressive symptoms and social support (n=489).

Variables	Participants	Depressive symptoms at follow-up			Social support at follow-up		
		Mean (SD)	*t* test (*df*), *F* test (*df*), or *r* value	*P* value	Mean (SD)	*t* test (*df*), *F* test (*df*), or *r* value	*P* value
Total, n (%)	489 (100)	10.74 (11.19)	—^a^	—	59.94 (14.97)	—	—
Age (years), mean (SD)	63.30 (13.35)		3.326 (487)^b^	<.001		−5.028 (487)^b^	<.001
Nonolder adult population	50.00 (8.82)	8.72 (9.48)			63.97 (14.08)		
The older adults population	72.36 (6.61)	12.12 (12.04)			57.20 (14.97)		
BMI at baseline (kg/m^2^), mean (SD)	23.99 (3.47)	—	−0.091 (487)^c^	.04	—	−0.034 (487)^c^	.46
BMI at follow-up (kg/m^2^), mean (SD)	23.89 (3.50)	—	−0.098 (487)^c^	.03	—	0.034 (487)^c^	.46
Sex, n (%)			−1.942 (487)^b^	.053		−1.307 (487)^b^	.19
Male	179 (36.61)	9.45 (9.56)			58.78 (14.92)		
Female	310 (63.39)	11.49 (11.99)			60.61 (14.99)		
Ethnicity, n (%)			1.122 (487)^b^	.26		0.079 (487)^b^	.94
Han	476 (97.34)	10.84 (11.27)			59.95 (15.05)		
Others	13 (2.66)	7.31 (7.47)			59.62 (12.39)		
Marital status, n (%)			−5.691 (487)^b^	<.001		2.061 (487)^b^	.04
Married	398 (81.39)	9.41 (9.77)			60.61 (14.63)		
Others	91 (18.61)	16.58 (14.70)			57.03 (16.16)		
Education, n (%)			12.069 (2)^d^	<.001		6.206 (2)^d^	.002
Illiteracy	209 (42.74)	13.54 (12.94)			57.34 (14.69)		
Primary school	137 (28.02)	9.07 (8.90)			60.90 (15.08)		
Middle school and above	143 (29.24)	8.27 (9.45)			62.82 (14.74)		
Occupation, n (%)			0.614 (487)^b^	.54		1.014 (487)^b^	.31
Farmers	311 (63.6)	10.98 (11.99)			60.46 (15.01)		
Others	178 (36.4)	10.33 (9.65)			59.03 (14.91)		
Mobile internet use at baseline, n (%)			−2.981 (487)^b^	.003		4.663 (487)^b^	<.001
Mobile internet users	110 (22.49)	7.96 (8.82)			65.68 (13.46)		
Others	379 (77.51)	11.55 (11.68)			58.27 (15.00)		
Social support at baseline, mean (SD)	59.15 (14.39)	—	−0.332 (487)^c^	<.001	—	0.263 (487)^c^	<.001
Depressive symptoms at baseline, mean (SD)	10.39 (10.59)	—	0.526 (487)^c^	<.001	—	−0.226 (487)^c^	<.001

a Not applicable.

b *t* test scores.

c *r* value.

d *F* test scores.

### Descriptive Data

Table 1 shows the results of these demographic characteristics and single-factor analysis. The results showed a positive correlation between age group (*t*_487_=3.326, *P*<.001), education level (*F*_2_=12.069, *P*<.001), baseline depression (*r*_487_=0.526, *P*<.001), and follow-up depression. BMI at baseline (*r*=−0.091, *P*=.04), BMI at follow-up (*r*=−0.098, *P*=.03), marital status (*t*_487_=−5.691, *P*<.001), MIU at baseline (*t*_487_=−2.981, *P*=.003), and social support at baseline (*r*=−0.332, *P*<.001) were negatively correlated with depression at follow-up. Marital status (*t*_487_=2.061, *P*=.04), education level (*t*_487_=6.206, *P*=.002), MIU at baseline (*t*_487_=4.663, *P*<.001) and social support at baseline (*r*=0.263, *P*<.001) were positively correlated with social support at follow-up. Age group (*t*_487_=−5.028, *P*<.001) and depressive symptoms at baseline (*r_487_*=−0.226, *P*<.001) were negatively correlated with social support at follow-up. All tests are 2-tailed as presented in Table 1.

### Main Results

#### Multivariate Analysis Based on LASSO Regression

**Figure 3.**
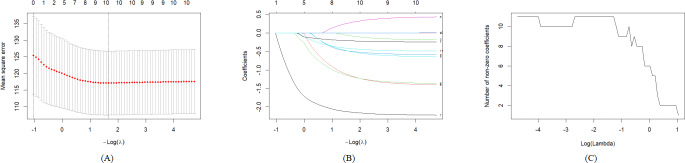
(A) Cross-validation error curve, (B) least absolute shrinkage and selection operator coefficient path diagram, and (C) relationship between non-zero coefficient quantity and λ for least absolute shrinkage and selection operator regression of model 1. LASSO: least absolute shrinkage and selection operator.

Based on the preset models 1‐3, variables are included in the LASSO regression model, and the best lambda value is selected as the screening criterion. According to Figures3^5^, significant variables are retained, respectively. In model 1, the non–older adult population, BMI at baseline, BMI at follow-up, male, ethnic Han, married, primary school, middle school and above, and mobile internet users are retained. In model 2, BMI at baseline, BMI at follow-up, male, ethnic Han, married, primary school, middle school and above, and social support at baseline are retained. In model 3, non–older adult population, BMI at baseline, male, married, occupation farmers, and mobile internet users are retained. The best lambda value and the coefficients under the best lambda value are presented in Table 2.

**Figure 4.**
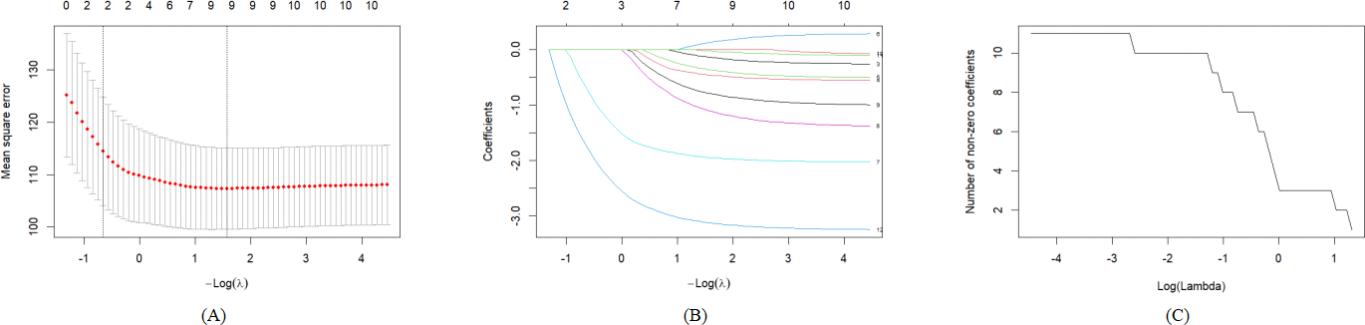
(A) Cross-validation error curve; (B) LASSO coefficient path diagram; and (C) relationship between nonzero coefficient quantity and λ forLASS0 regression of model 2. LASSO: least absolute shrinkage and selection operator.

**Figure 5.**
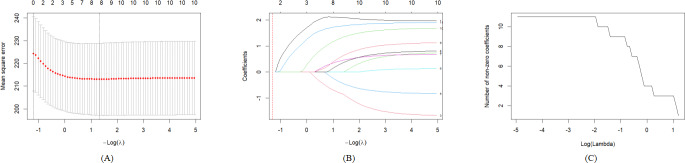
(A) Cross-validation error curve; (B)LASSO coefficient path diagram; and (C) relationship between non-zero coefficient quantity and λ for LASSO regression of model 3. LASSO: least absolute shrinkage and selection operator.

The robustness analyses demonstrated consistently high stability across all 3 models. For depression symptom predictors, bootstrap stability analysis revealed high selection frequencies for marital status (100%), primary education (97.4%), secondary education (96.3%), male (88.2%), and MIU (78.7%). In the social support mediation pathway, both baseline social support and marital status demonstrated perfect selection consistency (100%) across all bootstrap replications and maintained selection under all regularization conditions. Furthermore, MIU exhibited near-perfect selection stability (98.9%) in predicting social support. Sensitivity analyses confirmed consistent variable selection patterns across different regularization parameters for all key predictors, providing robust evidence for the reliability of the identified relationships.

**Table 2.** Least absolute shrinkage and selection operator regression analysis for the factors associated with social support and depressive symptoms.^a^

Variables	Model 1^b^	Model 2^b^	Model 3^c^
Constant	10.74233129	10.74233129	59.9399586
Age (years)			
The non–older adult population	−0.21301654	—^d^	2.1070368
The elderly population	—	—	—
BMI at baseline (kg/m^2^)	−0.07730491	−0.14305917	−0.8245169
BMI at follow up (kg/m^2^)	−0.49373459	−0.45496701	—
Sex			
Male	−0.5016278	−0.36233779	−0.4075946
Female	—	—	—
Ethnicity			
Han	0.28272745	0.12709935	—
Others	—	—	—
Marital status			
Married	−2.14018878	−1.94364746	0.4366721
Others	—	—	—
Education			
Illiteracy	—	—	—
Primary school	−1.15823898	−1.10337027	0.3243639
Middle school and above	−1.17625388	−0.7891779	0.5941248
Occupation			
Farmers	—	—	1.3140195
Others	—	—	—
Mobile internet use at baseline			
Mobile internet users	−0.42342155	−0.02666954	1.7695324
Others	—	—	—
Social support at baseline	—	−3.12424953	—
Best λ	0.1880534	0.2078466	0.2695602

a The numbers in the table represent the coefficients under the best λ value.

b dependent variable is depressive symptoms at follow-up

c dependent variable is social support at follow-up.

d Not applicable.

#### Cross-Lagged Model

Table 3 illustrates the correlation between MIU, social support, and depressive symptoms measured in the 2 periods. MIU, social support, and depressive symptoms were significantly correlated (all *P*<.001). This laid the foundation for us to establish a cross-lag model. The results of the correlation test are presented in Table 3.

**Table 3.** The correlation analysis of mobile internet use, social support, and depressive symptoms.

Variables	Value, mean (SD)	1	2	3	4	5
Mobile internet use at baseline	—^a^	—^b^	—^b^	—^b^	—^b^	—^b^
Mobile internet use at follow-up	—^a^	0.506^c^ (*P*<.001)	—^b^	—^b^	—^b^	—^b^
Social support at baseline	59.15 (14.39)	0.216^c^ (*P*<.001)	0.169^c^ (*P*<.001)	—^b^	—^b^	—^b^
Social support at follow-up	59.94 (14.97)	0.207^c^ (*P*<.001)	0.260^c^ (*P*<.001)	0.263^c^ (*P*<.001)	—^b^	—^b^
Depressive symptoms at baseline	10.39 (10.59)	−0.188^c^ (*P*<.001)	−0.187^c^ (*P*<.001)	−0.487^c^ (*P*<.001)	-0.226^c^ (*P*<.001)	—^b^
Depressive symptoms at follow-up	10.74 (11.19)	−0.134^c^ (*P*=.003)	−0.165^c^ (*P*<.001)	−0.332^c^ (*P*<.001)	−0.369^c^ (*P*<.001)	0.526^c^ (*P*<.001)

a Not applicable.

b The lower triangular part of the correlation matrix is presented. The upper triangular part is omitted as it is symmetric to the lower triangular part.

c *r* value.

**Figure 6.**
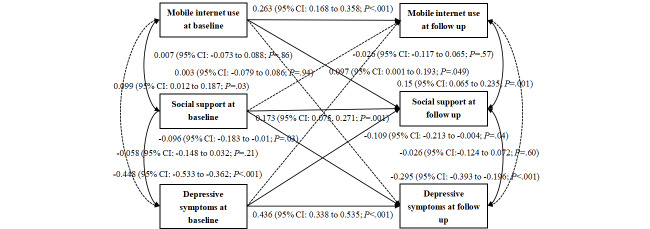
Output model of the cross-lagged panel model of mobile internet use, social support, and depression after adding control variables.

Figure 6 shows the significance of the relationship and path between the 3 variables in 2 periods. The relationship between baseline MIU and follow-up social support was significant (ρ=0.097, *P*=.049), and there was a 1-way relationship, indicating that baseline MIU had the same direction for follow-up social support. The relationship between social support and depressive symptoms is significant, and there is a significant bidirectional relationship. Baseline social support inversely predicts follow-up depressive symptoms (ρ=−0.096, *P*=.03), and baseline depressive symptoms also inversely predict follow-up social support (ρ=−0.109, *P*=.04). Baseline social support has a greater impact on follow-up depressive symptoms. The results of the cross-lagged model are illustrated in Figure 6. All parameters presented are standardized path coefficients.

## Discussion

### Key Results

This study delved into the longitudinal relationship between MIU, social support, and depressive symptoms among community residents, using cross-lagged modeling to track and analyze the potential interactions among the 3 during this 4-year period. The results indicated that MIU was significantly negatively correlated with depressive symptoms, suggesting that mobile Internet access and use may help alleviate depressive symptoms over time. Specifically, mobile internet appears to enhance social support and reduce depressive symptoms through several pathways; it helps users maintain and expand social connections, especially long-distance relationships, providing emotional support and a sense of belonging [22]. It offers platforms for accessing health information and joining interest communities, boosting self-efficacy and coping abilities; and positive internet-based interactions promote positive emotions and buffer stress [12,18,44]. Crucially, the mental health impact of mobile internet depends heavily on usage patterns: purposeful use for communication and learning often yields positive outcomes, whereas passive consumption or excessive use may increase psychological risks. Therefore, promoting healthy usage behaviors, not just access, should be a key focus for future digital health interventions.

Social support plays a longitudinal mediating role between MIU and depressive symptoms. Specifically, early mobile internet usage enhances later levels of social support through multiple behavioral and psychological mechanisms. By breaking temporal and spatial constraints, mobile internet enables individuals to more conveniently maintain long-distance relationships, access timely emotional support, and join internet-based communities based on shared interests, thereby significantly increasing their perceived social support [45]. This enhanced support system further alleviates depressive symptoms by providing emotional validation, reducing loneliness, and strengthening a sense of belonging [46]. Thus, while MIU does not directly reduce depression, it indirectly promotes mental health improvement by expanding and consolidating social support networks.

Higher levels of social support at baseline predicted significant reductions in depressive symptoms at subsequent follow-up. This phenomenon is consistent with extensive current research findings of a stable negative association between social support and depressive symptoms [26,47]. For the adolescent population, perceived social support is a key factor in preventing mental health problems and warding off the onset of depressive symptoms during the transition to adulthood [48]. In contrast, in the older population, research has shown that the impact of stressful life events on depressed mood in older adults can be effectively buffered by active participation in family and social activities, reducing the degree of mean difference [44]. In particular, lack of social support has been identified as a significant predictor of depressive symptoms in older men. In particular, lack of social support has been identified as a significant predictor of depressive symptoms in older men [11]. Notably, emotional support is widely recognized as a highly consistent and important protective factor against depressive symptoms in adults [49]. Thus, whether targeting adolescents, older adults, or the general population, enhancing social support networks is an effective strategy for alleviating depressive symptoms and promoting mental health.

Depressive symptoms at baseline similarly predicted lower levels of social support during the follow-up phase. This phenomenon can be explained through the perspectives of social behavior modification and the stress buffering model. From the social behavior modification angle, core depressive symptoms—such as persistent low mood, diminished interest, and cognitive deficits—reduce the frequency and quality of social interactions, thereby weakening the size and effectiveness of social support networks [50]. From the stress buffering model perspective, effective social support requires individuals to actively seek and accept it [51]. However, depression impairs both the willingness and ability to seek support, leading to isolation and reduced help-seeking. These behaviors erode the support system, as social support requires reciprocal interaction that becomes strained. Critically, depression distorts the perception of available support, diminishing its effectiveness. This creates a vicious cycle: diminished support exacerbates depression, which further weakens social connections.

Marital status and education significantly influence the relationship between social support and depressive symptoms. From a social capital theory perspective, marriage enhances resilience to stress by expanding individuals’ networks of strong ties (such as family and friends) and weak ties, thereby providing multidimensional support—emotional, instrumental, and informational—and reducing the risk of depressive symptoms [52]. Research suggested that the quality of marital intimacy negatively correlates with depression severity. Conversely, higher educational attainment often helps individuals cope more effectively with psychological stress by enhancing cognitive flexibility, promoting resource acquisition strategies, and boosting self-efficacy, thereby reducing depressive symptoms [53]. Education also indirectly promotes mental health by expanding social network diversity and elevating socioeconomic status. Consequently, marriage and education, as key social structural factors, play crucial roles in the mechanisms linking social support and depressive symptoms.

### Innovations

In this study, a longitudinal data design with 2 time points was adopted to enhance the reliability of inferring relationships between variables across both time periods. The application of the LASSO regression method for variable selection and regularization has improved the explanatory power and prediction accuracy of the model. Combined with cross-lag panel model analysis, this paper reveals the dynamic interaction mechanism between MIU, social support, and depressive symptoms.

### Limitations

This study has several limitations that should be considered. First, the reliance on self-reported questionnaires may introduce social desirability or recall bias. Second, there is no unified measurement standard for MIU, which may lead to reporting bias and limit the nuanced interpretation of usage patterns, as we did not quantitatively assess the duration, frequency, or specific behavioral characteristics of internet use. Third, although the sample size was sufficient for primary analyses, it may restrict subgroup analyses and generalizability, particularly given that the study focused solely on mobile internet access without including other digital devices (eg, desktops or laptops). In addition, while LASSO regression provides robust variable selection, its coefficient estimates do not permit traditional CIs due to inherent selection bias and nonstandard sampling distributions introduced by the regularization process. However, we addressed this limitation through comprehensive robustness checks, including bootstrap stability analysis and sensitivity testing across different regularization parameters, which demonstrated high consistency in variable selection. Finally, many potential influencing factors of depressive symptoms may not have been fully accounted for in the analysis. These limitations should be addressed in future studies.

### Conclusion

This study analyzed the relationship between MIU, social support, and depressive symptoms through longitudinal data. LASSO regression shows that social support plays a completely mediating role between MIU and depressive symptoms. The cross-lag model shows that MIU positively predicts social support, and there is a 2-way reverse predictive relationship between social support and depressive symptoms. These results reveal the complex impact of digital technology use on mental health, emphasizing the importance of promoting healthy internet-based social interactions for maintaining psychological well-being. In addition, it is recommended to develop digital tools that enhance real-life social connections and consider individual internet-based behavior patterns in mental health interventions. Future research should further explore the universality of this relationship across different cultural backgrounds in order to, first, develop more effective strategies for promoting mental health. Second, develop community-based digital literacy programs tailored to rural residents, focusing on how to use mobile internet for meaningful social interactions and reliable health information acquisition. Third, integrate internet-based social support enhancement into existing mental health interventions. For example, train primary health care providers to guide patients on using mobile platforms to maintain and strengthen social connections. Fourth, design public health campaigns that promote the conscious use of mobile internet for strengthening real-life social relationships, rather than passive or excessive consumption. Fifth, collaborate with telecommunications companies to improve network infrastructure in rural areas and develop affordable data plans, ensuring equitable digital access for mental health promotion. Finally, incorporate assessments of internet use patterns into routine mental health screenings in primary care settings to identify individuals at risk of problematic use and provide early intervention.

## Abbreviations

CES-D: Center for Epidemiological Studies-Depression Scale
LASSO: least absolute shrinkage and selection operator
MCAR: Missing Completely at Random
MIU: mobile internet use
STROBE: Strengthening the Reporting of Observational Studies in Epidemiology
